# Operation of NIST Josephson Array Voltage Standards

**DOI:** 10.6028/jres.095.026

**Published:** 1990

**Authors:** Clark A. Hamilton, Charles Burroughs, Kao Chieh

**Affiliations:** National Institute of Standards and Technology, Boulder, CO 80303

**Keywords:** Josephson array, Josephson junction, superconductivity, voltage standard

## Abstract

This paper begins with a brief discussion of the physical principles and history of Josephson effect voltage standards. The main body of the paper deals with the practical details of the array design, cryoprobe construction, bias source requirements, adjustment of the system for optimum performance, calibration algorithms, and an assessment of error sources for the NIST-developed Josephson array standard.

## 1. Introduction

### 1.1 Physical Principles

In 1962 Brian Josephson derived an equation for the superconducting tunnel current that flows through a thin insulating barrier separating two superconductors [1]:

$$I=I_{0}\sin\left(\frac{4\pi e}{h}\int V\;dt\right).$$ (1)

**Figure 1:**
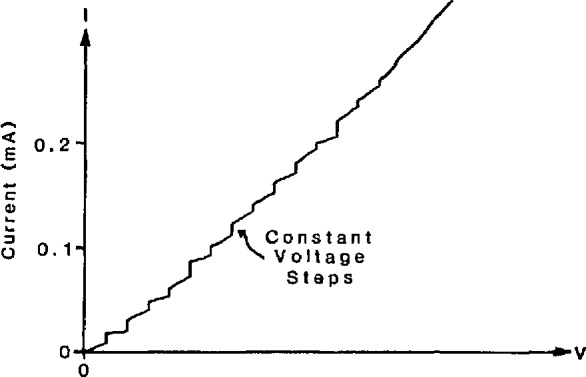
The *I-V* curve of a Josephson junction in a microwave field. The vertical steps are regions where the Josephson frequency 2*e*/*h* phase-locks to harmonics of the microwave frequency.

In this equation, *I* is the junction current, *I*_0_ is the critical current (a constant of the junction), *V* is the junction voltage, and *e*/*h* is the ratio of the elementary charge to Planck’s constant. When a dc voltage, *V*, is applied across the junction, eq (1) shows that the current will oscillate at a frequency *f* = 2*eV*/*h*, where 2e/*h* ≃ 484 GHz/mV. The very high frequency and low level of this oscillation make it difficult to observe directly. However, if an ac voltage at frequency *f* is applied to the junction, the junction’s self-oscillation has a strong tendency to phase-lock to the applied frequency. During this phase lock, the junction voltage must equal *hf*/2*e.* This effect, known as the ac Josephson effect, is experimentally observed as a constant-voltage step at *V = hf*/2*e* in the *I-V* curve of the junction. It is also possible for the junction to phase-lock to harmonics of *f*. This results in a series of steps at *V = nhf*/2*e*, as shown in figure 1.

### 1.2 History

Early experiments [2] showed that the Josephson voltage-frequency relationship implied by eq (1) is extremely precise. Initially these experiments used this relation to measure the ratio 2*e*/*h.* It was soon realized that such measurements were limited by the accuracy of the voltage measurements. Thus, in 1972, NIST (then the National Bureau of Standards or NBS) adopted the value 483 593.42 GHz/V for 2*e*/*h* for the purpose of measuring voltage. Since then the NBS/NIST Legal Volt has been based on the ac Josephson effect. The Josephson volt, however, is not a fundamental derivation of the volt but rather a means of providing a very stable and reproducible voltage reference. In the International System of Units (SI), the volt is derived from a number of experiments that relate electrical and mechanical units. When the above value for 2*e*/*h* was first adopted in 1972, the uncertainty in realizing the SI volt was about 10 parts per million (ppm). Since then this uncertainty has been considerably reduced. As a result, an international agreement has been reached to define the Josephson voltage by the equation *V = nf*/*K*_J_ with *K*_J−90_ = 483 597.9 GHz/V as the adopted value for the Josephson constant *K*_J_. The new definition was effective on January 1, 1990 [3–4].

Although the ac Josephson effect provides a better voltage reference than standard cells, the first Josephson standards were difficult to use, mainly because a single junction produces a very low voltage—from 1 to 10 mV. Early Josephson voltage standards were unique systems, custom-built for national standards laboratories.

The accuracy of Josephson voltage standards improves substantially when many junctions are connected in series to generate a large voltage. This approach has been used to achieve a level of 100 mV from 20 individually biased junctions [5]. Extension of this approach to larger voltages rapidly becomes impractical because it is necessary to bias each junction onto one of the constant voltage steps shown in figure 1.

**Figure 2:**
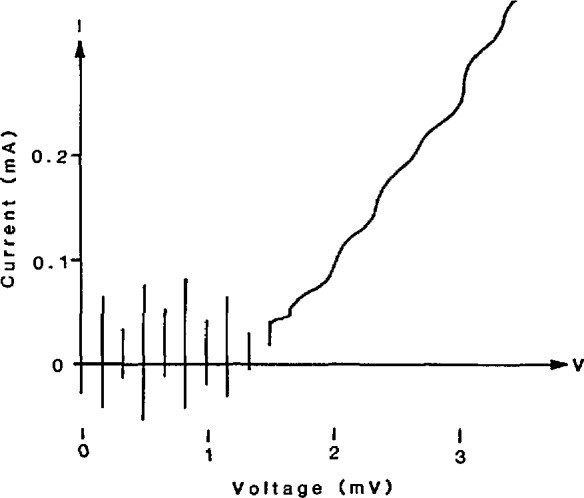
A single junction *I-V* curve showing constant voltage steps that cross the zero-current axis.

In 1977 Levinsen *et al.* suggested a method to avoid the multiple bias problem by using constant-voltage steps that cross the zero-current axis of the junction *I-V* curve [6]. These zero-crossing steps occur when highly capacitive junctions are exposed to microwave radiation at a frequency well above the junction’s natural resonant frequency. Figure 2 is an example of an *I-V* curve with zero-crossing steps; an important feature is the lack of stable regions between the first few steps. Thus for small bias currents, the junction voltage must be quantized. With a common bias current at or near zero, the voltage across a large array of these junctions must also be quantized. With arrays of up to 19 000 junctions, quantized voltages above 10 V are possible. After more than 10 years of effort, the problems of fabrication, stability, and rf distribution are largely solved, and Josephson array voltage standards are a reality in many laboratories around the world [6–18].

## 2. Josephson-Array Voltage Standards

### 2.1 Chip Layout

**Figure 3:**
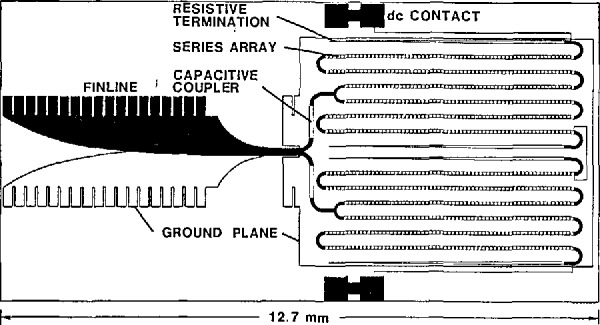
The layout of a typical Josephson-array chip.

The layout for a 1500-junction array is shown in figure 3. The rf-drive power is collected from a waveguide by a finline antenna, split four ways, and injected into four arrays of 375 series-connected junctions. The arrays are spaced 1 *µ*m above a superconducting ground plane. Since the junctions are highly capacitive, they have a very low impedance at the microwave frequency. The array thus acts as a microstripline. Typical attenuation is about 0.15 dB/mm. Matched loads terminate each Stripline so that each junction in the array receives the same level of rf drive. Addition of the dc voltages across the four arrays produces the I-V output. Capacitors prevent the dc voltage from shorting through the rf distribution network. The rf drive is applied by inserting the finline end of the chip into a slot parallel to the E-field in a WR-12 waveguide. The dc output appears across superconducting pads on the edge of the chip.

### 2.2 Operation

**Figure 4:**
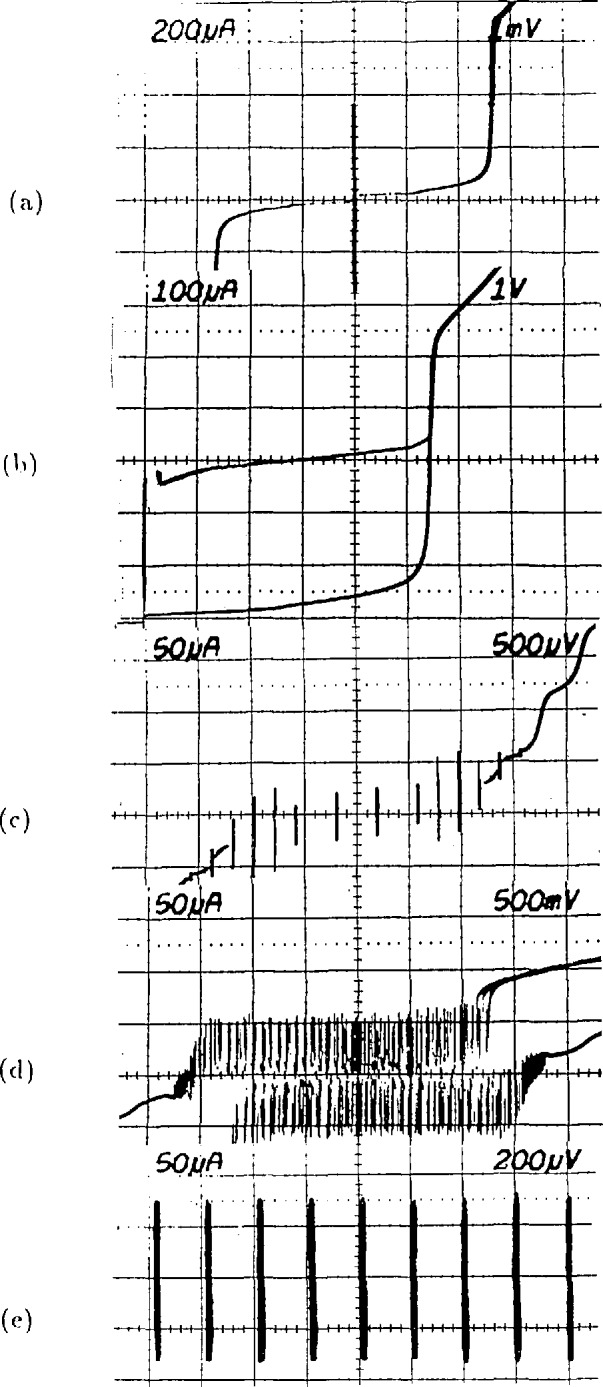
(a) A single-junction *I-V* curve, (b) an array *I-V* curve, (c) a single-junction *I-V* curve with applied rf, (d) an array *I-V* curve with applied rf, and (e) an expansion of curve (d) showing individual steps.

The operation of the array is illustrated in the *I-V* curves of figure 4. Figure 4a is a typical tunneling *I-V* curve for a single junction. This junction has a critical current of 360 *µ*A and an energy-gap voltage of 2.7 mV. Figure 4b is the *I-V* curve of 2076 of these junctions connected in a series array; addition of the energy-gap voltages of all the junctions produces a current rise at 5.5 V. The upper horizontal branch of the curve consists of 2076 transitions which occur as the junctions switch to the energy gap in the order of increasing critical current. This branch of the *I-V* curve shows that the critical-current distribution varies from 270 to 340 *µ*A for this array. When a 96-GHz current of about 15 mA is applied to a single junction, it develops constant-voltage steps in its *I-V* curve, as shown in figure 4c. Note that the steps below 1 mV cross the zero-current axis and that there are no stable regions between these steps. Figure 4d shows an *I-V* curve for the full array with 5 mW of rf drive (at the chip mount) at 96 GHz. This curve traces the envelope of about 15 000 constant-voltage steps that occur at 200-*µ*V intervals from −1.5 to +1.5 V. In figure 4e, a section of this curve near 0.7 V is expanded to show nine individual steps.

## 3. The Josephson-Array Voltage-Standard System

### 3.1 System Configuration

**Figure 5:**
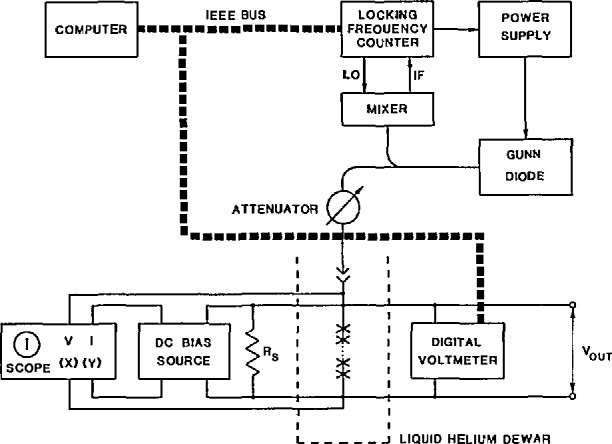
A block diagram of a Josephson-array voltage-standard system.

A block diagram of a typical voltage-standard system is shown in figure 5. The chip is mounted in a magnetically shielded cryoprobe and immersed in liquid helium. A Gunn diode, with an operating frequency in the range 70 to 100 GHz, provides microwave power to the chip. A microwave counter measures and phase-locks the Gunn diode frequency to an internal or external frequency reference. A dc-current source and a low-frequency triangle-wave generator provide current bias to the chip. The oscilloscope displays the bias current as a function of the array voltage. When the array is biased on a constant-voltage step, the oscilloscope displays a vertical line. If even one junction in the array is not biased on a step, the oscilloscope *I-V* display will have a slope which is easily detected on a suitably sensitive voltage scale, e.g., 10 *µ*V/div. The voltmeter and frequency counter are connected via the IEEE-488 bus to a computer that can perform calibration, recordkeeping, and diagnostic functions.

### 3.2 Cryoprobe

**Figure 6:**
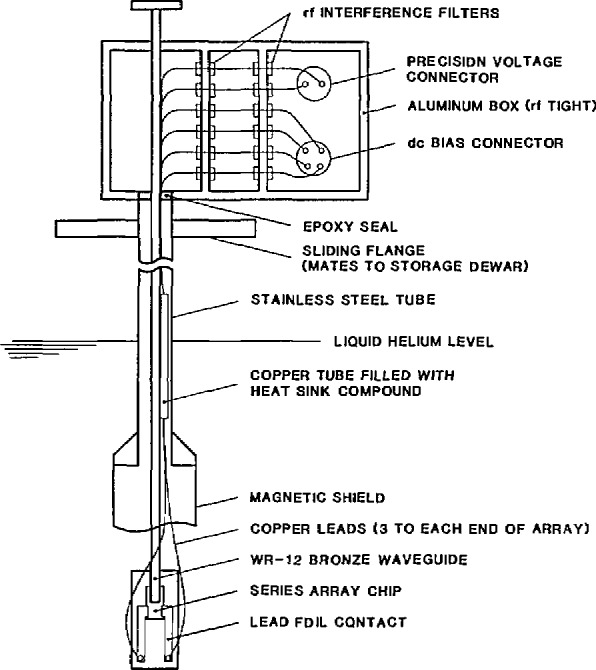
A typical cryoprobe used for cooling Josephson array voltage standards in a liquid-helium Dewar.

Figure 6 shows a typical cryoprobe on which Josephson-array voltage standards are mounted for cooling in liquid helium. Microwave power is delivered to the chip using a WR-12 band waveguide that has low-thermal and low-microwave loss. Typical microwave loss is 6 dB or less for a carefully cleaned waveguide. Dirt or corrosion in the waveguide can increase the attenuation by as much as 10 dB. A suitable waveguide can be made by using either a continuous section of 90–10 bronze or two sections of coin silver waveguide separated by 15 cm of internally gold-plated stainless steel waveguide. The latter must be constructed so that the stainless steel section will be located in the neck of the Dewar when the probe is fully submerged in the helium. The waveguide is plugged at the chip mount flange by a tapered dielectric plug or a thin polyethylene sheet between the flanges. The plug prevents thermal helium oscillations from developing inside the guide.

Although much more expensive, a dielectric waveguide can achieve an attenuation as low as 2 dB and negligible thermal loss. This makes it possible to keep the cryoprobe continuously immersed in a 100-L storage Dewar for as long eight weeks between refills.

To prevent noise-induced transitions between constant voltage steps, the array chip must be completely covered by an rf and magnetic shield, and all connections to the chip must be through RFI filters at the probe top. These filters should have a minimum attenuation of 60 dB above 100 kHz and 100 dB above 1 MHz. If the array is to be connected directly to a DVM, it may be necessary to add additional low-frequency filtering to the reference output leads. To prevent ground loops, the microwave source and mixer must be isolated from the probe with a dc break that is made by removing the pins and separating the waveguide flanges at the top of the cryoprobe with a thin layer of polyethylene. The flanges are attached with nylon screws.

To minimize thermal voltage errors, the probe should be wired with thermocouple-grade copper wire that is bound to the waveguide with plumber’s tape. A total of six wires, three to each side of the array, is typically used. Four wires are used to bias the array and to display its *I-V* curve; the other two wires supply the precise voltage output. The thermal voltage on the precise voltage leads may be checked by shorting the leads at the bottom end of the probe and measuring the voltage at the top connector as the probe is lowered into a Dewar of liquid helium. The voltage change after cooling should be less than 1 *µ*V. Since there are six wires to the sample mount, performance can often be improved by selecting the pair with the smallest thermal voltage.

### 3.3 Bias Circuit

**Figure 7:**
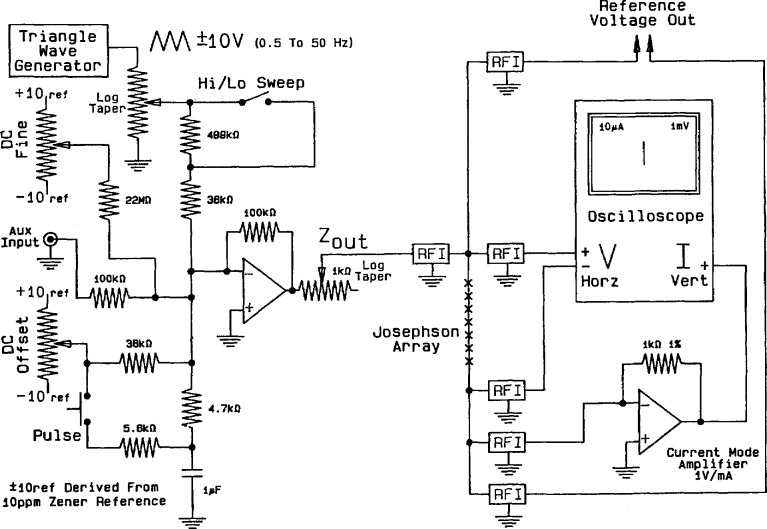
A circuit for supplying bias current to a Josephson array.

A suitable bias circuit, shown in figure 7, provides an output bias that is the sum of a triangle-wave sweep and a dc offset. The dc offset must be stable and adjustable at a level of about 200 *µ*V. The output impedance is controlled by a 1-kΩ log-taper potentiometer. A pulse button provides the capability to momentarily increase the dc offset by about a factor of 3. This is often useful in achieving high-level, stable, array voltages. The bias current is measured by detecting the return current at the virtual ground input of a current mode amplifier. This means that current flowing into the RFI filter capacitance is not measured. The result is an *I-V* curve display (fig. 4) without the hysteresis caused by the filter capacitance and the associated distortion. The horizontal amplifier on the oscilloscope should have a differential input with a sensitivity of at least 100 *µ*V. If this is not available, a differential preamplifier should be used.

### 3.4 Grounding and Ground Leakage

Grounding of the array circuit at only one point (usually the return current side of the bias supply) is essential. In any case, most oscilloscopes have an input impedance to ground of not more than 1 MΩ. Thus, anything connected to the reference voltage output must be completely floating (more than 10^10^ Ω to ground). If there is a ground leakage path, the resulting current will cause voltage drops in the probe wiring resistance that lead to unpredictable errors. These errors are not detected or canceled by usual reversal procedures. A procedure for measuring leakage in the cryoprobe is described in section 6.4. Ground leakage and ac noise in reference standards can be eliminated by operating them in battery mode with line power completely disconnected. Leakage in the digital voltmeter (DVM) can be detected by performing calibrations with the DVM both at ground potential and at the array potential (1 V). If there is leakage in the DVM, these measurements will not agree.

## 4. Array Operation

### 4.1 Cooling and Warming

The probe should be cooled by lowering it slowly (over a period of about 15 min) into a liquid-helium Dewar. There should be no connections to the probe during the cooling process. The bias connections must be checked to ensure that the bias voltage is set to within 2 mV of 0 V. Once the probe is fully immersed in the helium and the bias level is verified, the bias leads can be connected. Failure to follow this procedure may lead to a number of problems caused by trapped magnetic flux in the array as described in section 4.2.

When the cryoprobe is removed from the Dewar, the Josephson array must be carefully warmed to avoid condensation on the chip. Any condensation on the chip or trapped inside the shield may lead to premature chip failure. Another approach is to store the probe with the chip mount raised into the neck of the Dewar. This is very safe for the chip but increases the chance of condensation in the waveguide. Unless a dielectric waveguide is used, storing the chip immersed in the liquid helium may cause excessive helium loss due to the thermal conductivity of the metal waveguide.

### 4.2 Array *I-V* Curve

**Figure 8:**
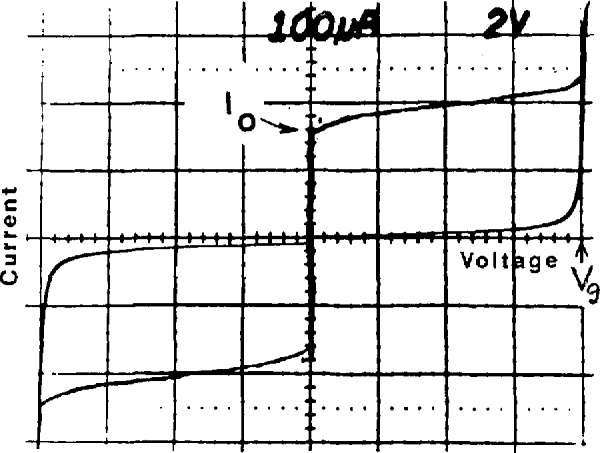
The full *I-V* curve of a typical Josephson array.

Successful operation of a Josephson array demands careful adjustment of the dc and rf bias controls. The first step in the tuning procedure is to sweep the array *I-V* curve over a voltage range which includes the combined energy gap voltage of all of the junctions. This is typically ±10 V for a 3000 junction array. (For very large arrays designed to reach 10 V, there is usually a maximum voltage limit which will prevent observation of the combined energy gap voltage.) A source impedance of 1 kΩ or greater produces the best display. With the microwave power turned off, the curve should be like that in figure 8. The mean value of the critical current, *I*_0_, and the combined energy-gap voltage, *V*_g_, should be recorded each time the chip is cooled. If *V*_g_ is lower than expected, the array may be partially shorted or it may not be at the 4.2-K operating temperature due to insufficient liquid helium. Changes in *I*_0_ may indicate moisture damage.

**Figure 9:**
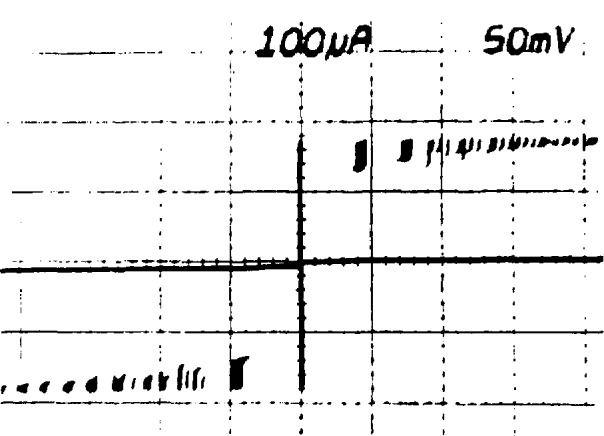
A typical array *I-V* curve with no trapped magnetic flux.

**Figure 10:**
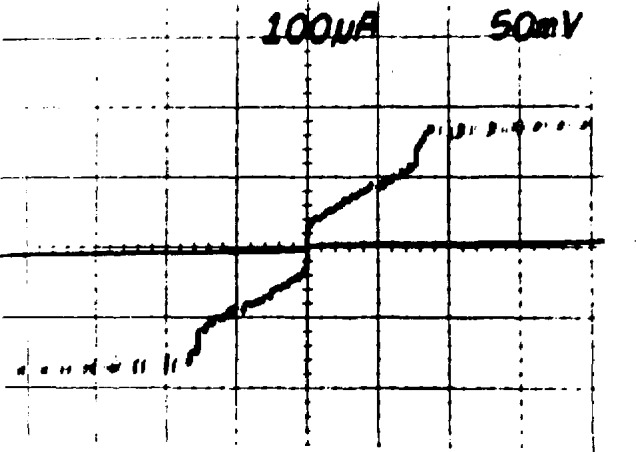
An array *I-V* curve with reduced critical currents caused by trapped magnetic flux.

The array must be checked for trapped magnetic flux or junction failures. These problems can be observed by increasing the voltage sensitivity to 50 mV/div. The lowest critical current should not be less than about 60% of the mean critical current, as shown in figure 9. One or more junctions with small critical currents will result in small and unstable steps in the array *I-V* curve. Figure 10 is an example of an *I-V* curve in which about 35 junctions have reduced critical currents due to trapped magnetic flux. This usually is caused by cooling the array with the bias leads connected or by connecting the bias leads when the bias voltage is not set to zero. Trapped flux can be removed by raising the probe to warm the chip above the transition temperature (as indicated by a linear *I-V* curve), disconnecting all leads to the array, and then recooling. If a low critical current is not cured by this process, it is probably caused by a junction failure. Such a failure will prevent the array from producing stable steps.

### 4.3 Optimizing the Power and Frequency

**Figure 11:**
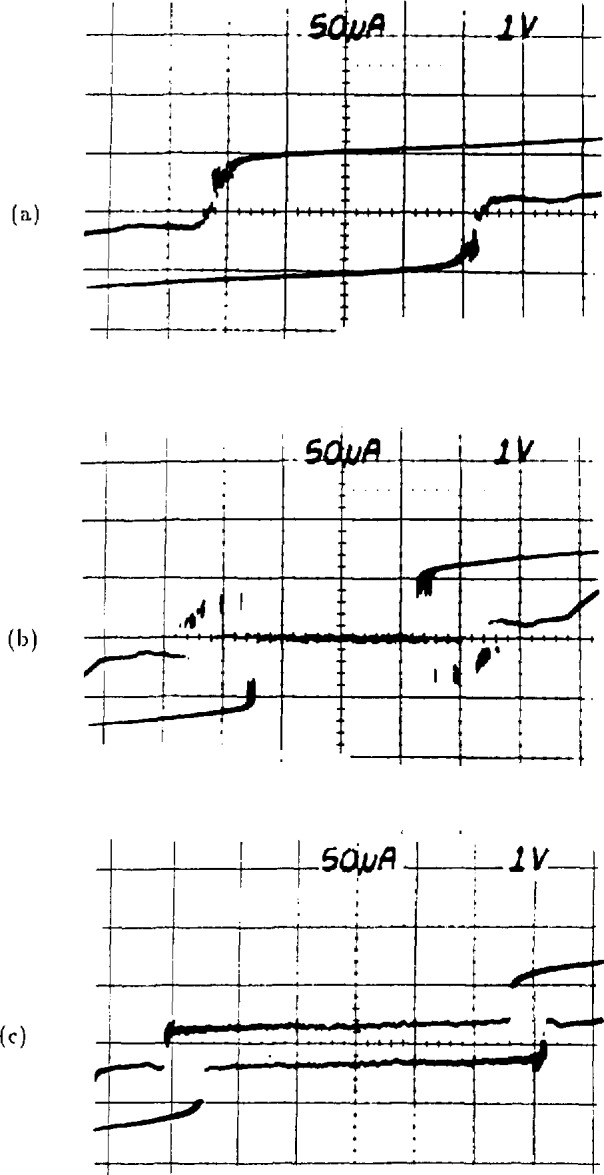
Typical array *I-V* curves for (a) low, (b) medium, and (c) high rf power levels.

**Figure 12:**
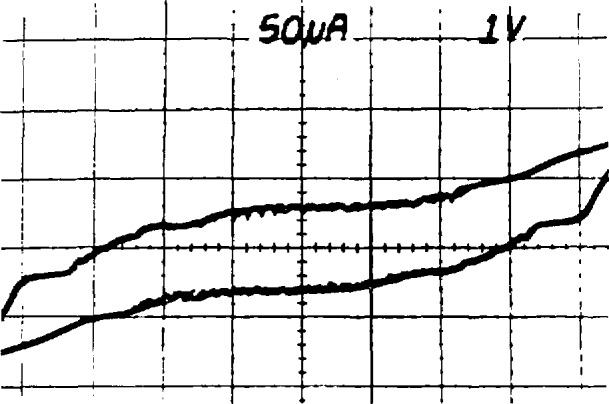
An array *I-V* curve showing the effect of too much rf power.

**Figure 13:**
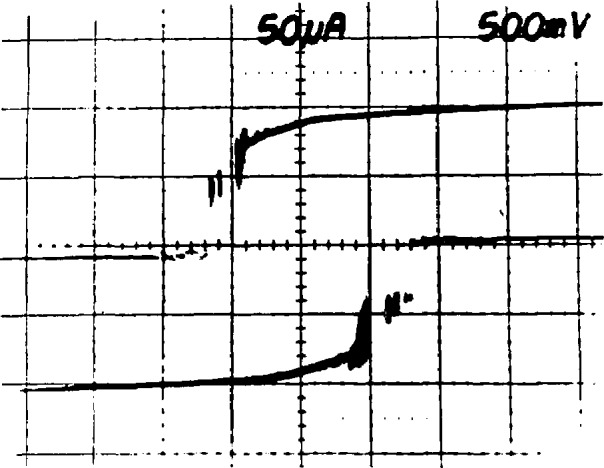
An array *I-V* curve showing the effect of too little rf power.

Proper operation of a Josephson-array voltage standard requires the selection of a frequency where the array and microwave source work well together. The microwave power and frequency must be adjusted to obtain an *I-V* curve like those shown in figure 11. The important features of these curves are (1) they encircle the desired operating voltage on the voltage axis, *e.g.*, 1 V, and (2) the center part of the upper and lower branches of the curve are reasonably flat. The area encircled by these curves is an indication of the voltage range over which stable steps can be found. From top to bottom, the three curves show how the *I-V* curve evolves as the power is increased. Figure 11a illustrates stable steps to about 2 V; figure 11b, to about 2.5 V; and figure 11c, to about 3 V. The vertical spread of the curve can depend on the source impedance and sweep rate; it does not necessarily indicate the step amplitudes. Figure 12 shows the array *I-V* curve when the microwave power is too high. In this case, the steps are small and unstable. In figure 13, the microwave power is too low to generate stable steps above about 0.5 V. The best way to set the frequency is to adjust the power to obtain a curve like that in figure 13 (steps to about 0.5 V) and then tune the frequency to maximize the width encircled on the voltage axis. The power can then be set to obtain a result like that in figure 11.

### 4.4 Setting the Array Voltage

**Figure 14:**
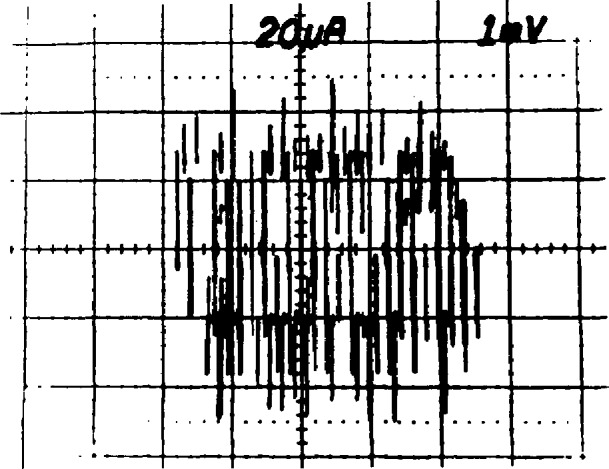
An ideal *I-V* curve display of quantum voltage steps.

**Figure 15:**
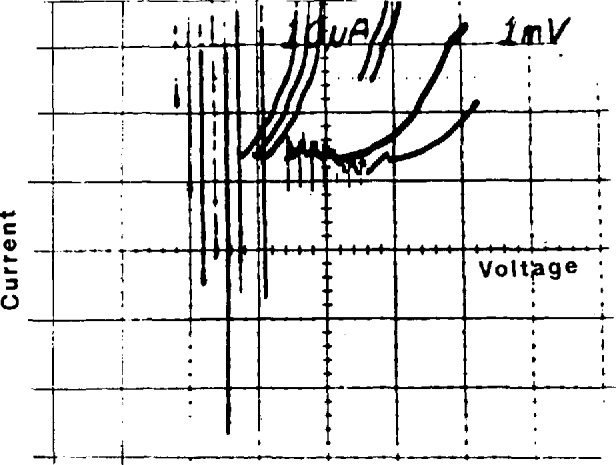
An expanded array *I-V* curve showing the effect of too little power.

**Figure 16:**
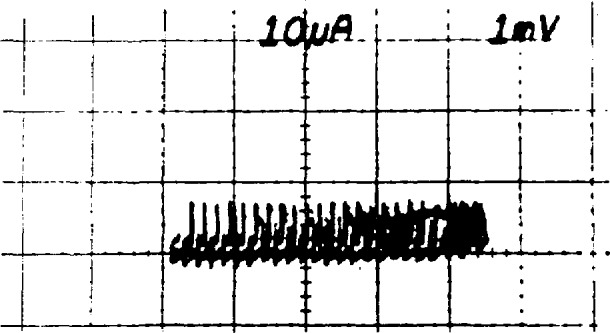
An expanded array *I-V* curve showing the effect of too much rf power.

**Figure 17:**
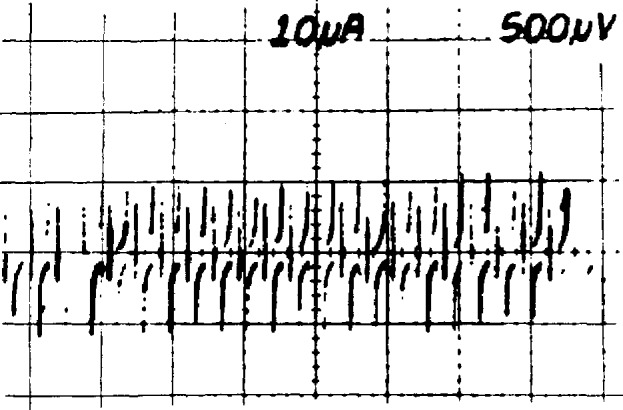
An expanded array *I-V* curve showing the effect of trapped magnetic flux.

When the array is used to calibrate a secondary reference, it is desirable to match the array and reference voltages as closely as possible. This is done by choosing appropriate values of *n* and *f* in the array voltage equation *V*_a_ *= nf*/*K*_J_. The value of *f* is set by the frequency stabilization loop of the microwave source. The bias supply is used to force the array to the proper quantum voltage integer, *n* as follows: Once the frequency and power have been set as described above, the bias impedance is set to about 20 Ω, and the dc offset is set to force the array to a voltage near the desired final value. The *I-V* curve should then be observed with 1-mV sensitivity by using either ac coupling or a dc offset on the voltage amplifier. Figure 14 shows the ideal result: a small ac sweep causes the array to switch among about 25 steps. The power can now be fine tuned to obtain maximum step heights. In general, the steps grow larger and become unstable as the power is reduced below the optimum value. A typical result when the power is too low is shown in figure 15. Although the steps are very large, some junctions are being biased onto resistive portions of their *I-V* curves as seen in the sloped regions on the right side of the figure. If the power is too high, the steps are small and unstable, with sloped regions between them, as shown in figure 16. If there is trapped magnetic flux, the steps will be small at all power levels. Figure 17 shows a situation in which the *I-V* curve of a single junction with trapped flux appears at many points along the voltage axis. The changing offset of this curve is caused when other junctions in the array switch between steps.

Once the optimum power and frequency adjustments have been made, a single quantum voltage step is selected by reducing the ac sweep to about 10 *μ*a. With a 15-Ω source impedance, any one of about six steps is selected at random. There are several ways to force the array to a specific step. For most arrays, an effective method is to reduce the sweep and bias impedance to minimum values. This may result in enough current noise to cause the array to switch among a few steps. The dc offset should then be adjusted to make the average array voltage as close as possible to the desired value, usually within 0.2 mV. When the bias impedance is increased, the array is very likely to lock onto the desired step. If increasing the bias impedance causes the bias point to move away from the *I = 0* axis, severe ground leakage probably exists somewhere, and any measurements are questionable. Some arrays go into a resistive state when the bias impedance is reduced. This may indicate that the operating frequency or power should be adjusted or that the array itself is marginal. Momentarily pulsing the bias voltage to a few volts above the desired value or turning the microwave power to zero may put the array back into the quantized voltage state.

Another method of selecting steps is to set the bias impedance to about 20 Ω and the sweep level to cause switching among about 25 steps, as in figure 14. Then set the dc offset to center the display on the desired step. When the sweep amplitude is reduced, the array is likely, after a few tries, to lock onto the desired step. Once the desired step is found, increasing the bias impedance to about 1 kΩ reduces the bias source current noise and improves the step stability.

## 5. Calibrations with an Array System

The most common use of a Josephson array is the calibration of a secondary reference standard, usually a Zener diode. Weston cells can be directly calibrated but the possibility of current transients caused by switching in the array makes this a risky procedure. Calibrations are typically done by putting the array and secondary reference in series opposition and measuring the difference voltage with a sensitive DVM. If the DVM has a gain error, *E*_g_, its contribution to the calibration error will be *E*_g_ (*V*_a_ − *V*_r_). Thus, the error will be minimized by matching the array voltage, *V*_a_, to the reference voltage, *V*_r_, as closely as possible. Since the quantum voltage number, *n*, and the frequency, *f*, can be set, it is possible to obtain a nearly perfect null, thus eliminating any contribution from the DVM gain error. However, modern eight-digit DVMs are so accurate that it is not usually necessary to match *V*_a_ and *V*_r_ to less than a few millivolts. This means that any frequency can be used and any quantum step within a few millivolts of null is sufficient. This relaxation of the null requirement necessitates occasional calibrations of the DVM against the array but substantially simplifies the calibration procedure.

To find the array voltage, *V*_a_*=nf*/*K*_J_, it is necessary to determine the frequency, *f*, and the step number, *n.* The frequency is determined by phase-locking the microwave source to an accurately known frequency standard. The step number, *n*, is calculated from a knowledge of the difference voltage, *V*_dvm_ and an estimate of the reference voltage, *V*_e_, as follows: Assume that the DVM is connected so that *V*_dvm_ = *V*_a_ *− V*_r_. Then *n* is given by

$$n=\text{Round}\left[\left(K_{J}/f\right)\left(V_{\text{dvm}}+V_{e}\right)\right],$$ (2)

where “Round” means rounded to the nearest integer. The array voltage can then be calculated in the usual way; that is, *V*_a_ *= nf*/*K*_J_. This procedure yields the correct value of *n* and *V*_a_ as long as the estimate *V*_e_ is within 50 *μ*V of the actual reference voltage, *V*_r_.

This procedure is more complicated than measuring the array voltage directly with the DVM, but it is much more convenient because it is not necessary to switch the array output to the DVM terminals. Such switching often induces a change in the step number, *n.*

In practice, there are always thermal voltages and offsets in the zero point of the DVM; these lead to errors in the measured difference voltage, *V*_dvm_. If the offset and thermal voltages are stable, the resulting errors can be eliminated by making a second measurement in which the reference voltage and quantum number, *n*, are reversed. When these results are averaged, the thermal and meter-offset voltages cancel. A third measurement can be used to correct for first-order drift in the thermal and meter-offset voltages. More complex procedures involving further reversals of the reference or the DVM generally do not significantly improve the results.

### 5.1 Calibration Algorithm

**Figure 18:**
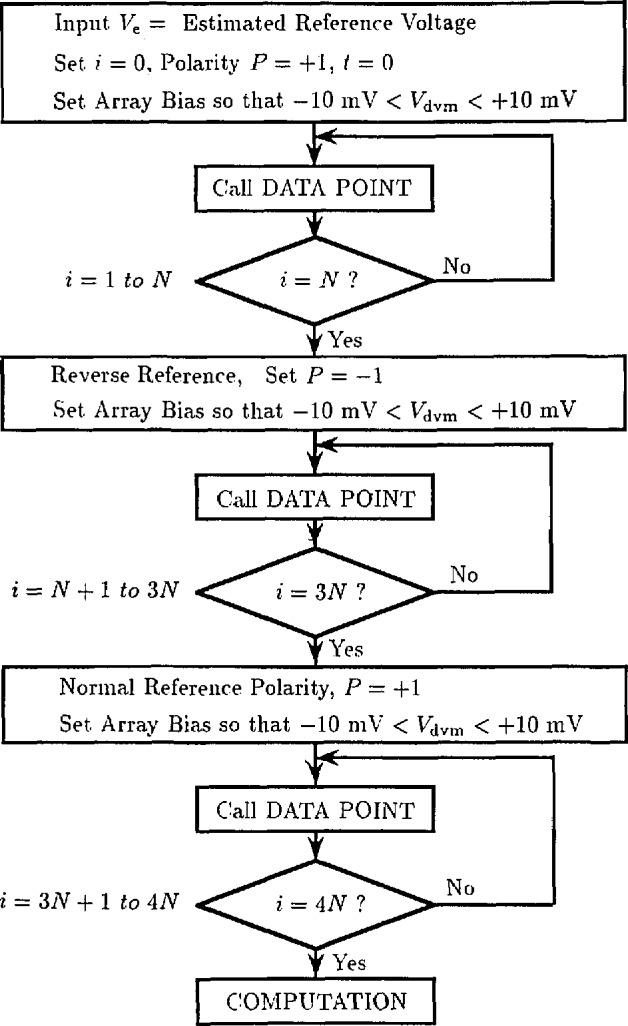
The flow chart for an algorithm used to calibrate a reference standard against a Josephson array.

The flow charts in figures 18 through 20 show a typical computer algorithm for calibrating reference standards. This algorithm has the following features:

1. The final uncertainty is controlled by a parameter, *N*, that specifies the number of difference readings to be averaged.
2. Any of about a hundred steps near the null point may be used for the measurement.
3. Spontaneous switching between steps is automatically accounted for during the measurement.
4. Frequency drift is tolerated with a corresponding increase in uncertainty.
5. The DVM and thermal offsets and first-order drifts are fully compensated.

The algorithm assumes that the DVM has a gain error, *ϵ*, less than 10 ppm. The DVM and thermal offsets and drifts are modeled by a combined offset voltage, *v*_0_, and drift rate, *m*. The array and reference are first connected in series opposition so that

$$V_{\text{dvm}}≃V_{a}-V_{r}+V_{0}+mt,$$ (3)

where *t* is the elapsed time since the first measurement. The array voltage, *V*_a_, is then adjusted within a few millivolts of *V*_r_ and values of *V*_dvm_ are obtained. Each value is checked for consistency (±2 *μ*V) with the two previous values and then used to compute a data point,

$$V_{i}=V_{a}-V_{\text{dvm}}.$$ (4)

The elapsed time, T*_i_*, is also recorded. The consistency check rejects readings affected by step transitions and enables a good data set to be obtained even when the array makes occasional transitions between steps. After *N* values of *V_i_* and *T_i_* have been obtained, the reference voltage and array bias are reversed and *2N* more values of *V_i_* and *T_i_* are obtained. Finally, *N* more values of *V_i_* and *T_i_* are obtained with the original configuration. Figure 21 shows a typical data set obtained using *N* = 10.

**Figure 20:**
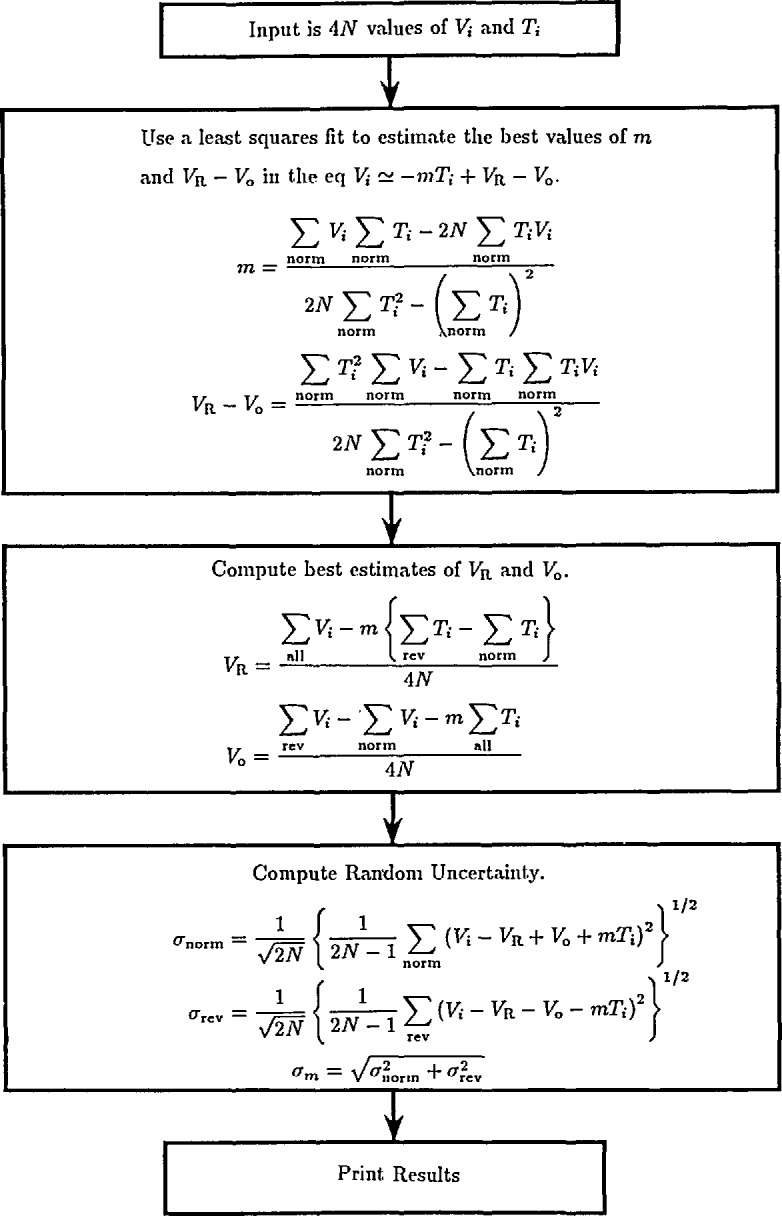
A flow chart of the calculation used to correct the calibration for thermal and DVM offsets and their first-order drifts.

In the computation flow chart of figure 20, the summation signs refer to data obtained with normal and reverse reference polarity and have the following definitions:

$$\sum_{\text{norm}}=\sum_{i=1}^{i=N}+\sum_{i=3N+1}^{i=4N}\;,$$ (5)

$$\sum_{\text{rev}}=\sum_{i=N+1}^{i=3N}\;,$$ (6)

$$\sum_{\text{all}}=\sum_{i=1}^{i=4N}\;.$$ (7)

**Figure 21:**
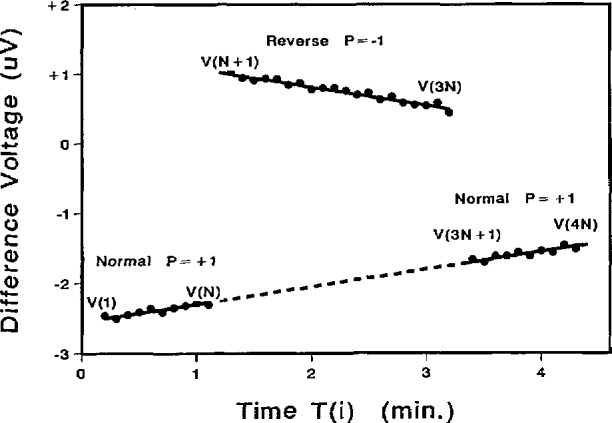
The data accumulated in a typical calibration of a reference standard.

A least-squares algorithm is used to compute values of *V*_r_ *− V*_0_ and *m* that fit the *2N* normal-polarity data points, *V_i_* and *T_i_*, with minimum error. This establishes the offset and drift line shown in figure 21. Reverse-polarity data are then used to estimate *V*_r_ and *V*_0_.

The uncertainty due to reference and DVM noise is calculated from the differences between the measured data and the linear fit of figure 21. A standard deviation of the mean, *σ*_norm_, is calculated for the normal data and *σ*_rev_ for the reverse data. The uncertainty in the value of *V*_r_ is calculated as the root-sum-square of *σ*_norm_ and *σ*_rev_.

It is very important to reverse the array voltage by reversing the bias rather than by using a reversing switch. Any procedure that does not reverse the array quantum number, *n*, will not cancel the approximately 1-*μ*V thermal voltage in the probe wiring.

## 6. Error Budget

Proper use of a Josephson-array voltage standard requires a careful assessment of all significant sources of error. This section develops an error budget appropriate to calibration of a 1-V reference standard that uses the calibration algorithm shown in figures 18 through 20. Both random and systematic errors must be considered. Random errors are obvious because they result in day-to-day fluctuations in calibration results. Even if the origin of the fluctuation is not known, the resulting uncertainty can be determined from a statistical analysis of the data. Systematic errors are more subtle because they generate a fixed or slowly drifting offset from the true value. Estimating the magnitude of systematic errors generally requires some special measurement procedure.

### 6.1 Difference-Voltage Measurement Noise

The dominant error in calibrations performed with a properly tuned Josephson voltage standard is the random noise of the difference-voltage measurement. Noise in the DVM and the reference standard combine to generate an uncertainty in the final calculation of the reference value. This random error, *E_n_*, is computed as shown in the algorithm of figure 20. Typical Zener reference calibrations at the 1-V level, using about one-minute averaging times for each of the normal and reverse measurements, should have 1*σ E_n_* uncertainties of about 0.02 ppm.

### 6.2 DVM Gain and Linearity Errors

In a typical reference calibration with a Josephson standard it is inconvenient and unnecessary to match the array and reference voltages exactly. Thus, any error in the difference-voltage measurement results in an error in the final reference value. In addition to noise, DVMs have linearity and gain errors. At the low levels typically used in the difference-voltage measurement, the DVM linearity-error specification is a fixed percentage of full scale, and the actual error typically fluctuates randomly from one digital code to the next [18]. As a result of noise, the difference-voltage measurement is averaged over many digital codes. Thus, at the low level of the difference measurement, linearity errors are indistinguishable from DVM noise and are automatically included in *E*_n_.

If the DVM gain, *G*, is not exactly 1, the error in the difference voltage is (*G* − 1)*V*_dvm_, and this leads to an error, *E*_d_, in the reference value, *V*_r_, given by

$$E_{d}=\left(G-1\right)\frac{V_{\text{dvm}}}{V_{r}}$$ (8)

For example, if the difference voltage is 1 mV and *G* = 1.000 010 (10 ppm error), the resulting error in a 1-V calibration is 0.01 ppm. The principal sources of uncertainty in *G* are the uncertainty in the initial DVM calibration and drift in the internal DVM voltage reference. Thus, the gain error, *E*_g_, can be written as a time-scaled quantity,

$$E_{g}=G-1=E_{c1}+D_{v}t,$$ (9)

where *E*_c1_ is the DVM calibration uncertainty, *D*_v_ is the drift-rate specification of the DVM, and *t* is the elapsed time since the last calibration. The term *E*_g_ in eq (9) can be substituted for (G − 1) in eq (8) to give

$$E_{d}=\left(E_{c1}+D_{v}t\right)\frac{V_{\text{dvm}}}{V_{r}}.$$ (10)

For a good-quality DVM, *D*_v_ is 10 ppm per year or less. This specification generally applies just after a self-calibration. If the DVM is subjected to the self-calibration daily, has its internal reference calibrated monthly, and the difference voltages are kept below 10 mV, then *E*_d_ will not add significantly to the total reference calibration uncertainty.

**Figure 22:**
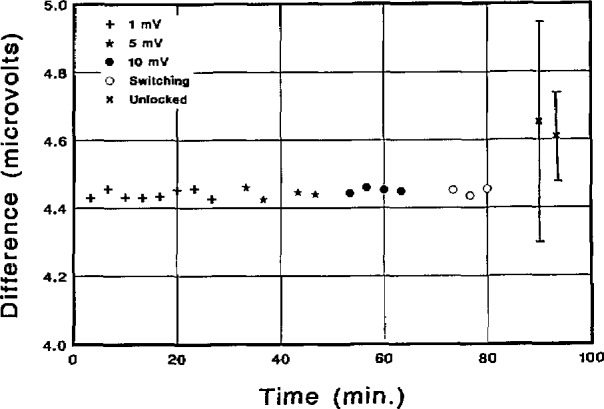
Calibration results of a 1.018-V Zener reference standard showing the effects of the difference-voltage amplitude, step stability, and frequency stability.

This point is illustrated in figure 22, which shows five sets of consecutive calibrations of a 1.018-V Zener reference standard all performed within 2 h. In the first three sets, the reference and Zener voltages were matched within 1, 5, and 10 mV. Since there is no apparent correlation between the reference values and the difference voltage, we can conclude that, with the DVM used in this measurement, difference voltages up to 10 mV can be tolerated without introducing significant errors. The fourth set of data in figure 22 shows three calibrations in which the array was deliberately forced to switch between steps many times during the measurement. This slows the measurement somewhat but has very little effect on the final result. The 1*σ* uncertainty on the first four data sets is just slightly larger than the points themselves. This uncertainty results from a combination of Zener and DVM noise. In this case it is dominated by noise in the 1-V Zener reference.

The last two calibrations were made with the frequency source unlocked. The frequency typically drifted 5 ppm during these measurements. However, since the frequency is measured for each data point, the effect of the drift on the final result is much less than 5 ppm. Since the frequency and voltage are measured at slightly different times, frequency fluctuations result in an increase in the random noise of the difference voltage, *E*_n_. Calibrations performed in the unlocked mode have an uncertainty that is highly dependent on the frequency stability. A typical value is about 0.3 ppm.

### 6.3 Frequency Error

Any error in the frequency reference that is used to measure and stabilize the microwave source translates directly into a voltage error. Josephson-array standards commonly rely on a precise quartz crystal frequency reference that is periodically calibrated. In this case, the fractional systematic error contributed by the frequency reference is

$$E_{f}=E_{c2}+D_{f}t,$$ (11)

where *E*_c2_ is the error in the initial calibration, D_f_ is the drift specification of the crystal, and *t* is the elapsed time since the frequency calibration. For example, if *E*_c2_ = 10^−10^ and *D*_f_ = 2 × l0^−10^ per d, about three months after a frequency calibration, the frequency error is comparable to the noise error.

In an automated system, time-scaled errors *E*_f_ and *E*_d_ should be incorporated in the calibration results so that their effects cannot be ignored.

### 6.4 Leakage Currents

Significant errors may occur if there is leakage current between the array voltage leads or from these leads to ground. Leakage current causes a voltage drop across the resistance, *R*_p_, of the probe filters and results in an error that is not corrected by the reversal process. Two kinds of leakage must be considered: resistive leakage and dielectric absorption. Resistive leakage can result from fingerprints on the filter elements, solder flux, or faulty capacitor dielectric. Dielectric absorption is a time-dependent process caused by a slow change in the orientation of electric dipoles in the filter elements.

**Figure 23:**
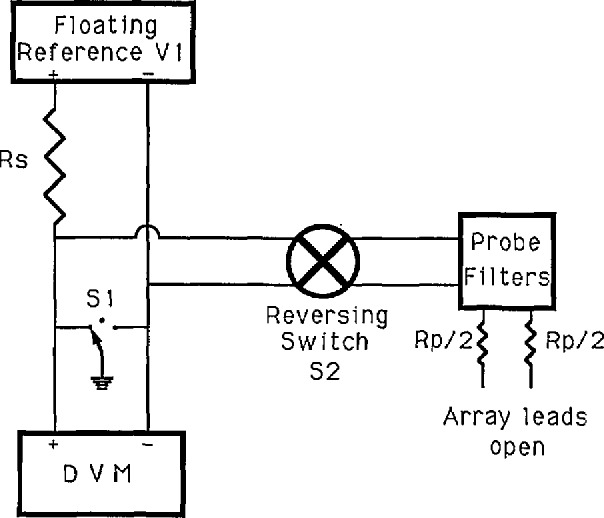
A circuit for measuring leakage currents in the cryoprobe.

Both resistive leakage and dielectric absorption sources may be checked with the circuit shown in figure 23. When a large resistance, *R*_s_ (typically 200 kΩ), is placed in series with the reference standard, the effects of leakage currents are magnified about 10 000 times. With the reversing switch S2 open, the DVM reads the reference voltage. If the DVM reading changes as a function of the position of switch S1, then ground-leakage current probably exists in the DVM. If the change is more than 10 *µ*V, the DVM should be repaired or replaced.

**Figure 24:**
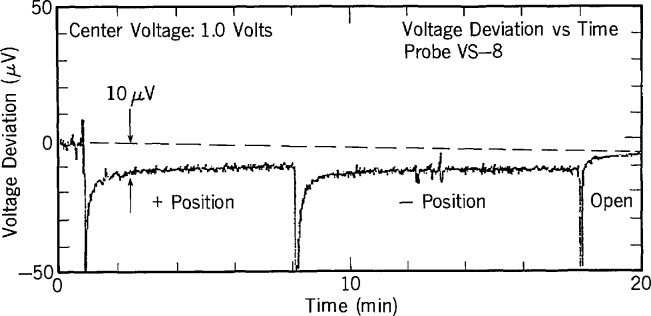
A plot of the DVM voltage vs. time generated using the circuit of figure 23. This plot is used to measure leakage and dielectric absorption in the cryoprobe.

When S2 is closed, any leakage current in the probe wiring causes an easily detected voltage drop in the DVM reading. A time plot of the DVM reading is shown in figure 24, first with the switch open, then in the (+) position, and finally in the (−) position. During these measurements, motion around the apparatus must be minimized because it induces capacitive currents that add considerable noise to the signal.

The systematic error due to cryoprobe leakage is calculated as follows: Let the deviation of the DVM voltage from the open switch voltage be Δ*V.* The leakage current is then given by *I*_L_ = Δ*V*/*R*_s_. When the probe is used with an array, this leakage current generates a normalized error,

$$E_{1}=\frac{I_{L}R_{p}}{V_{r}}=\frac{\Delta VR_{p}}{V_{r}R_{s}},$$ (12)

where *V*_r_ is the reference voltage used in the leakage-current measurement. It is convenient to choose *R*_s_ = 10^4^ *R*_p_ since this makes Δ*V* exactly 10^4^ times the actual error voltage. Figure 24 is an example of the result of this procedure for a probe with *R*_p_ = 20 Ω and *R*_s_ = 200 kΩ. The large spikes at the reversal points are caused by the current required to charge the filter capacitors. The exponential response is due to dielectric absorption and demonstrates that at least 20 s is required between a reversal and the beginning of a difference voltage measurement. The offset of about 10 *μ*V from the open-switch voltage indicates that the error due to probe leakage is about 10 *μ*V/10^4^ = 1 nV. Most of this leakage is contributed by the filter elements. For the specific results shown in figure 24, this error does not contribute significantly to the total uncertainty.

Leakage current in the leads connected to the reference standard will result in a voltage drop across the internal resistance, *R*_r_, of the standard. The fractional error in the reference voltage is *R*_r_/*R*_1_ where *R*_1_ is the leakage resistance. Since *R*_r_ is typically about 1 kΩ, leakage resistance greater than 10^12^ Ω is required to keep this error below 1 part in 10^9^. This can be easily achieved by using an appropriate reversing switch, insulated wire, and by floating the reference standard. The error caused by the leakage resistance *R*_1_ can be evaluated with the same procedure used to determine the probe leakage error as described above. Leakage current errors should be checked a few times a year and every time the precise voltage wiring is changed.

### 6.5 Uncorrected Thermal Offsets

Any thermal voltages in the wiring between the reversing switch and the reference standard are not corrected in the reversal process described in section 5. For this reason, very low thermal wire should be used and the distance between the reversing switch and the reference standard should be as short as possible. Uncorrected thermal voltages may be divided into a constant part and a part that may change with each switch operation. Both parts can be estimated by replacing the reference source with a low thermal short and connecting the DVM to the reversing switch output. The DVM should be set for a long averaging time so that voltages of a few nanovolts can be resolved. A series of *n* ≃ 10 DVM readings should then be taken to determine the 1*σ* uncertainty due to DVM noise, *σ*_dvm_. If this is more than a few nV, then a longer DVM averaging time should be selected. Next, a series of 2*n* measurements with alternating switch polarities should be recorded. Let these be called *V_+i_* and *V_−i_, i* = 1…*n.* If the 1*σ* values for *V_+i_* and *V_−i_, σ_+_, σ_−_*, are significantly greater than *σ*_dvm_, then there is a fractional error *E*_r_ due to switch repeatability given by

$$E_{r}=\frac{1}{V_{r}}\sqrt{{\left(\frac{\sigma_{+}+\sigma_{-}}{2}\right)}^{2}-\sigma_{\text{dvm}}^{2}}.$$ (13)

The offset voltage, *V*_w_ contributed by the wiring is given by

$$V_{w}=\frac{1}{2n}\sum_{i=1}^{l}V_{+i}-V_{-i}$$ (14)

with an uncertainty given by

$$\Delta V_{w}=\sqrt{\frac{1}{n}\left(\sigma_{+}^{2}+\sigma_{-}^{2}\right)}.$$ (15)

The resulting fractional error in *V*_r_ is

$$E_{w}=\Delta V_{w}/V_{r}.$$ (16)

If *V*_w_ is significant relative to Δ*V*_w_ it should be subtracted from the reference voltage *V*_r_. *E*_w_ should then be included in the combined uncertainty. Uncorrected thermal voltages are typically less than 10 nV, which is near the detection threshold.

There still remains the possibility of an error due to thermal voltages in the short or its connections. This error cannot be determined because any procedure to measure it requires altering the connections which may be the source of the error. Since it cannot be measured, this error is generally ignored.

### 6.6 Combined Uncertainty

If the previously described uncertainties are uncorrelated, the total 1*σ* uncertainty is obtained by adding them in quadrature:

$$E_{\text{tot}}=\sqrt{E_{n}^{2}+E_{d}^{2}+E_{f}^{2}+E_{l}^{2}+E_{r}^{2}+E_{w}^{2}}.$$ (17)

## 7. Common Problems

### 7.1 Inadequate Microwave Power

The frequency response of typical microwave sources and Josephson arrays is not very flat. Therefore, the power coupled into the array can often be substantially increased by tuning the source.

If the array response indicates that the available power is lower than expected from experience, it is likely that moisture has condensed in the waveguide. In this case, both the chip mount and microwave source must be removed from the probe and warm dry gas blown through the waveguide for about 5 minutes.

### 7.2 Erratic Calibration Results

A common mode of chip failure is the appearance of a slope on the *I-V* curve of the quantum-voltage steps. A slope occurs when a resistance develops in any part of the array circuit, usually as a result of corrosion or physical damage. Sloped steps can easily produce a scatter of up to 10 ppm in calibration results. Since this slope may have a value of 0.1 Ω or less, the maximum available voltage sensitivity (typically 10 *μ*V) must be used to search the *I-V* curve for this problem.

If the polarity of the array, reference, or DVM does not conform to the assumptions made in the calibration algorithm, unpredictable errors up to about 70 ppm will occur in the calculated reference value.

The calibration algorithm computes the array step number based on an estimate of the reference voltage. If this estimate is in error by more than about 70 ppm, the computed step number will be wrong and the calibration result will therefore be meaningless.

### 7.3 Unstable Steps

Frequent switching between steps is, perhaps, the most common problem encountered with Josephson-array voltage standards. Good stability requires proper adjustment of the bias level, bias impedance, and microwave power, as discussed in section 4.3. Noise pick-up in the wires connected to the array can easily cause spontaneous transitions between the quantized voltage levels. Such noise is typically caused by switching of high-power equipment and by local radio transmitters. The “walkie-talkies” often used by service personnel can be a particularly uncontrollable and unpredictable source of interference. Usually, interference problems can be substantially reduced by careful shielding and grounding. In most environments, a shielded room is not necessary.

If the array is not fully immersed in liquid helium, it is likely to generate unstable steps. This possibility is easily checked by comparing the array energy-gap voltage, *V*_g_, with its nominal value. A reduction of more than 5% indicates inadequate cooling.

Nonuniform critical currents in the array junctions also lead to unstable steps. This may be caused by trapped magnetic flux or the failure of one or more junctions. If the problem is not cured by warming and recooling the array, then a junction failure is the probable cause, and the array must be replaced. When care is taken to avoid physical damage and exposure to moisture, and thermal cycling is minimized, typical arrays have a lifetime of several years or more.

## Figures and Tables

**Figure 19:**
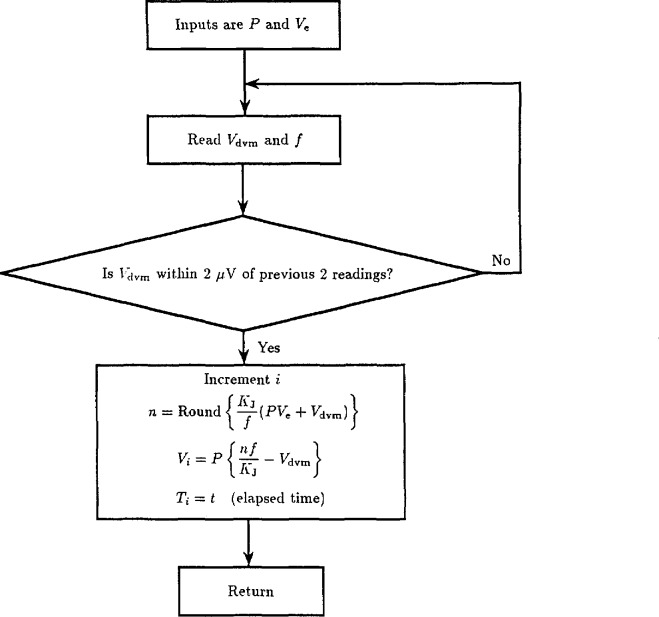
The flow chart for a subroutine used to obtain a single measurement of the reference voltage.
